# Supplementary material for: Combined Effect of tDCS and Motor or Cognitive Activity in Patients with Alzheimer’s Disease: A Proof-of-Concept Pilot Study
Source: Brain Sci. 2024 Oct 30;14(11):1099. doi: 10.3390/brainsci14111099 (PMC11591620; doi:10.3390/brainsci14111099)
Supplement: Supplementary file 1 [file brainsci-14-01099-s001.zip › Figure S1_Graph Changes in MMSE scores from baseline (T0) to post-treatment (T1) and fol-low-up (T2).pdf]

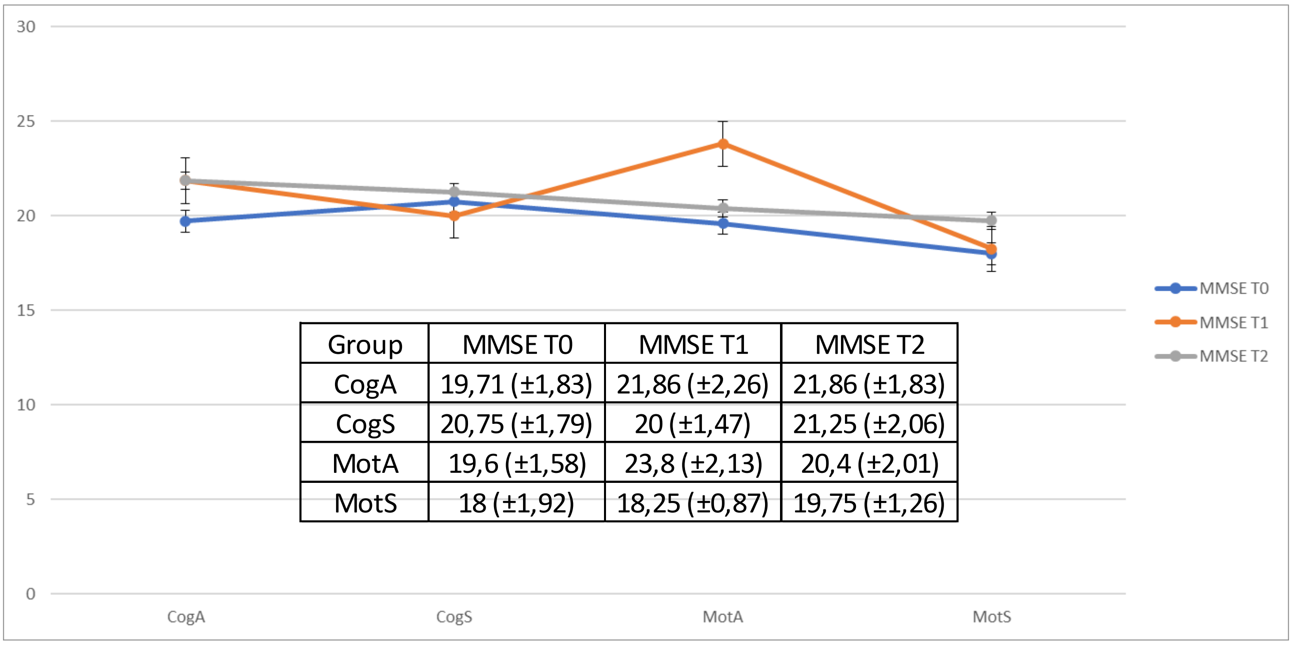

Group	MMSE T0	MMSE T1	MMSE T2
CogA	19,71 (±1,83)	21,86 (±2,26)	21,86 (±1,83)
CogS	20,75 (±1,79)	20 (±1,47)	21,25 (±2,06)
MotA	19,6 (±1,58)	23,8 (±2,13)	20,4 (±2,01)
MotS	18 (±1,92)	18,25 (±0,87)	19,75 (±1,26)

Graph. Changes in MMSE scores from baseline (T0) to post-treatment (T1) and follow-up (T2). Data are given as mean ± standard error. Abbreviations: MotA: Motor activity + Anodal stimulation; MotS: Motorial activity + Sham stimulation; CogA: Cognitive activity + Anodal stimulation; CogS: Cognitive activity + Sham stimulation, MMSE: Mini Mental State Examination. We have provided the means and standard errors for each group and evaluation time in the table linked to the graph.
