# Supplementary material for: Combined Effect of tDCS and Motor or Cognitive Activity in Patients with Alzheimer’s Disease: A Proof-of-Concept Pilot Study
Source: Brain Sci. 2024 Oct 30;14(11):1099. doi: 10.3390/brainsci14111099 (PMC11591620; doi:10.3390/brainsci14111099)
Supplement: Supplementary file 1 [file brainsci-14-01099-s001.zip › Figure S2_Graph Changes in VRT scores from baseline (T0) to post-treatment (T1) and fol-low-up (T2).pdf]

Group	T0 VST	T1 VST	T2 VST
CogA	32,71 (±6,90)	39,14 (±6,83)	32,85 (±6,35)
CogS	32,5 (±6,86)	35 (±6,70)	33,25 (±5,13)
MotA	27,2 (±12,80)	31,6 (±9,50)	26,4 (±11,63)
MotS	32,75 (±7,10)	29,5 (±7,70)	34,5 (±8,10)

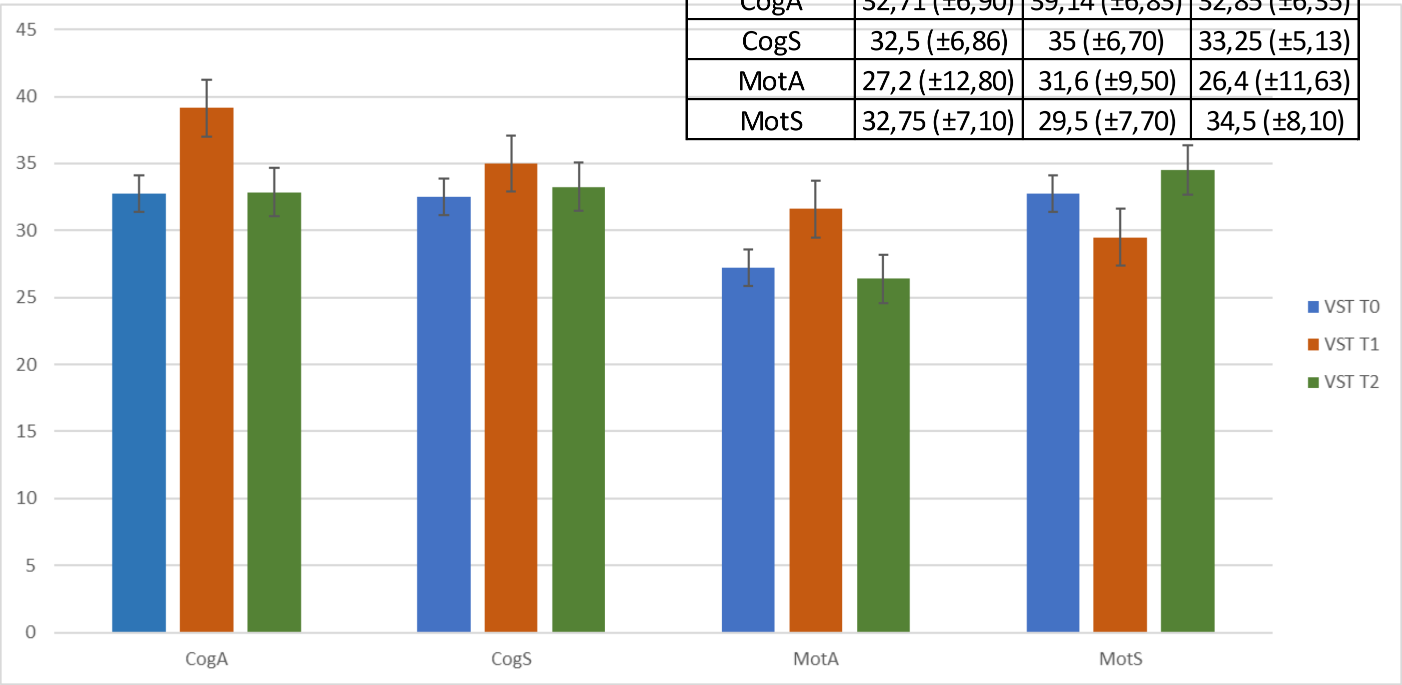

Graph. Changes in VST scores from baseline (T0) to post-treatment (T1) and follow-up (T2). Data are given as mean ± standard error. Abbreviations: MotA: Motor activity + Anodal stimulation; MotS: Motorial activity + Sham stimulation; CogA: Cognitive activity + Anodal stimulation; CogS: Cognitive activity + Sham stimulation, VST: Visual Search Test. We have provided the means and standard errors for each group and evaluation time in the table linked to the graph.
