# Supplementary material for: Combined Effect of tDCS and Motor or Cognitive Activity in Patients with Alzheimer’s Disease: A Proof-of-Concept Pilot Study
Source: Brain Sci. 2024 Oct 30;14(11):1099. doi: 10.3390/brainsci14111099 (PMC11591620; doi:10.3390/brainsci14111099)
Supplement: Supplementary file 1 [file brainsci-14-01099-s001.zip › Figure S3_Graph. Changes in FA-SART scores from baseline (T0) to post-treatment (T1) and follow-up (T2).pdf]

Group	FA SART T0	FA SART T1	FA SART T2
CogA	10,57 (±1,93)	8,66 (±0,86)	12 (±2,39)
CogS	3 (±2,21)	10,5 (±2,02)	9,5 (±2,82)
MotA	7,25 (±1,52)	7,2 (±2,95)	8,2 (±4,26)
MotS	5 (±1,15)	10,25 (±3,92)	7,75 (±3,32)

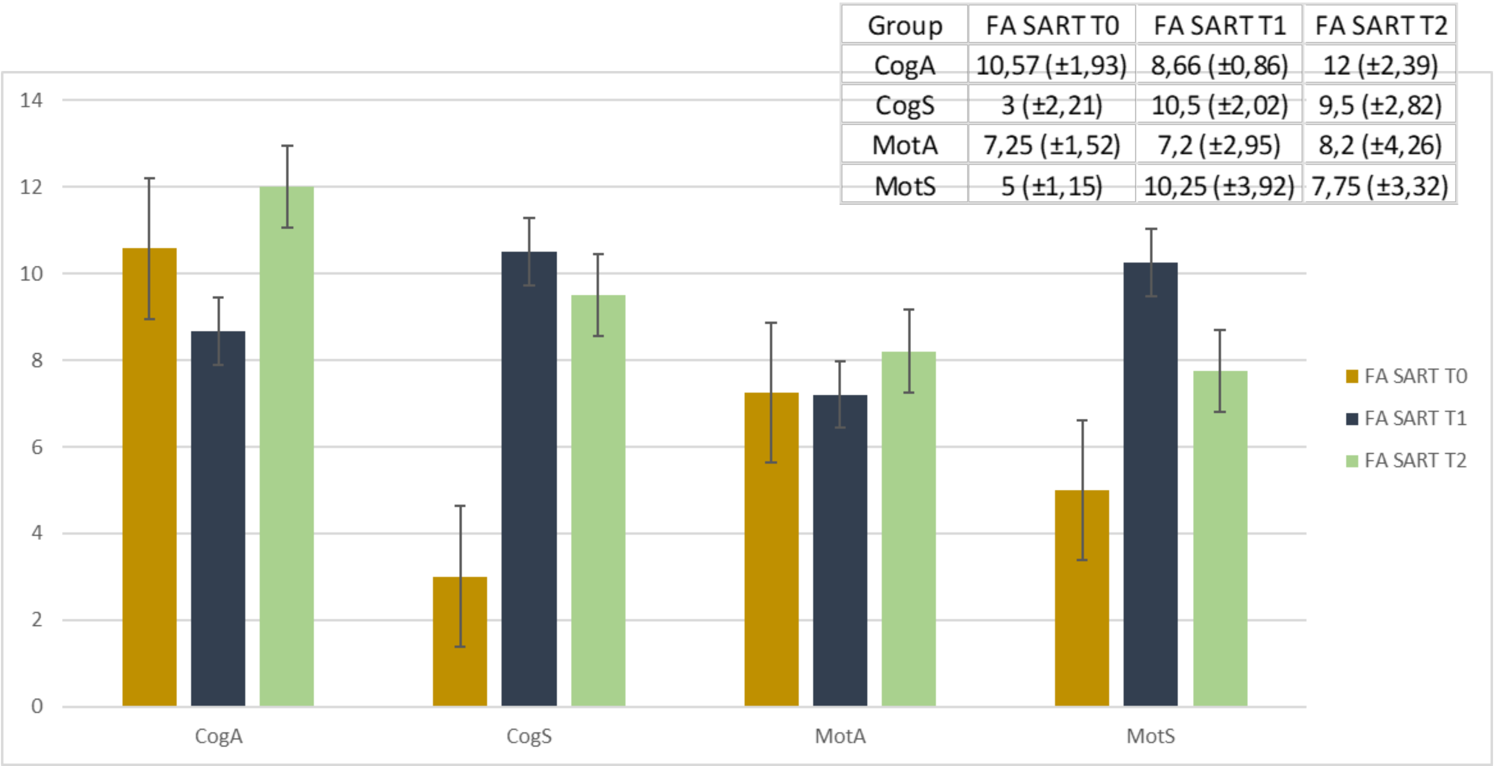

Graph. Changes in FA-SART scores from baseline (T0) to post-treatment (T1) and follow-up (T2). Data are given as mean ± standard error. Abbreviations: MotA: Motor activity + Anodal stimulation; MotS: Motorial activity + Sham stimulation; CogA: Cognitive activity + Anodal stimulation; CogS: Cognitive activity + Sham stimulation, FA-SART: Sustained Attention to Response Test-False Allarm (FA). We have provided the means and standard errors for each group and evaluation time in the table linked to the graph.
